# Look on the bright side: the relation between family values, positive aspects of care and caregiver burden

**DOI:** 10.1007/s10433-024-00819-9

**Published:** 2024-08-30

**Authors:** Larissa Zwar, Hans-Helmut König, André Hajek

**Affiliations:** Department of Health Economics and Health Services Research, University Medical Center Hamburg-Eppendorf, 20246 Hamburg, Germany

**Keywords:** Familism, Informal caregiver, Family care, Burden, Stress, Positive aspects of care, Resilience

## Abstract

Family-centered values are important for caregiving. However, findings on their association with burden are inconsistent. We aim to analyze whether positive aspects of caregiving are mediating the effect of familism on burden among informal caregivers of older adults in Germany. Participants (*n* = 277) were drawn from the Attitudes Toward Informal Caregivers (ATTIC) project and include informal long-term caregivers of older relatives (aged ≥ 60) quota-sampled from Germany (December 2023). Mediation analyses (linear OLS regression) with robust standard errors were conducted with the classic and the counterfactual causal mediation framework. The classic approach indicated a significant positive direct effect of familism on burden, a significant negative direct effect of PAC on burden and a significant negative indirect effect of familism via PAC on burden; the total effect was not significant. The causal mediation approach supports this; the interaction between familism and PAC was not significant. Thus, sociocultural family-centered values seemed to worsen burden but also to reduce it through positive experiences of caregiving, which did not depend on the strength of familism values. The findings advance our understanding of the mechanisms underlying the stress appraisal of the informal care situation and emphasize the role of positive experiences of care.

**Supplementary Information:**

The online version contains supplementary material available at 10.1007/s10433-024-00819-9.

## Introduction

A growing group of young and older adults are stepping up to provide unpaid care for individuals with care needs—mostly for their older relatives (aged 65 years and older, (Federal Statistical Office 2020; Statistisches Bundesamt 2020)). These informal caregivers are critical to meet the increasing demand for care due to ongoing demographic aging, as is reflected in care policy in countries like Germany, which gives precedence to home care supported by informal caregivers (Federal Ministry of Justice 1994). However, informal caregiving for older relatives can be very stressful and result in worse wellbeing and (mental) health (Bom et al. 2019; Zwar et al. 2018, 2020, 2023), making it essential to identify and modify factors that influence their burden of care.

### The appraisal of caregiving

According to the transactional stress model and the care-specific stress appraisal model (Lazarus and Folkman 2020; Verbakel et al. 2018), the caregivers’ appraisal of a situation as stressful (i.e., burdensome (Graessel et al. 2014)) or beneficial (Verbakel et al. 2018) is based on personal and contextual resources and demands or barriers (see Supplementary Material, Figure A1). If demands outweigh resources, for example, a full-time employed caregiver providing several time-consuming care tasks but having a low level of social support, stress is experienced, which can impact health and wellbeing negatively (Lazarus and Folkman 2020). These influential factors of the appraisal process, acting as resources or barriers, can include internalized factors, such as sociocultural values resulting from the cultural context (Knight and Sayegh 2010; Lazarus and Folkman 2020). In particular, family-centric values, i.e., familism values, are expected to be relevant for the appraisal process and the consequences of the informal care situation for older relatives.

### Familism

Familism is a multidimensional collectivistic construct, primarily developed with Latino and Hispanic caregiver populations (López-Anuarbe et al. 2022; Losada et al. 2020; Steidel and Contreras 2003), but found across cultures (Schwartz 2007). It refers to an individual’s strong connection and commitment to their family members and can be divided into attitudinal, structural and behavioral familism (Sabogal et al. 1987). In this paper, we focus on the attitudinal aspect of familism, which refers to values about support, interconnectedness, obligations and loyalty to the family (Christophe and Stein 2022; Steidel and Contreras 2003). These familism values are highly relevant for informal care as caregivers often name these values as a reason for becoming a caregiver, in particular for relatives (Zarzycki et al. 2023). Placing more importance on family interconnectedness and support may help to reduce the perceived burden among informal caregivers. Namely, perceiving care more in line with and fulfilling one’s own values could change the appraisal of caregiving to be less negative and stressful and thus result in lower burden. However, this is not supported unequivocally by previous findings. While some research points to family-centered values as protective factors (Guo et al. 2019; Gupta et al. 2009; Maximiano-Barreto et al. 2022), other research raises doubts by finding no significant association between familism and burden at all, neither in terms of familial obligation nor with family support (Tian et al. 2022). Adding to this, familism was shown to be associated with worse mental health (Tian et al. 2022) and dysfunctional thoughts (Losada et al. 2010). Further research is therefore needed to understand the mechanism between both, familism and burden.

Hence, this study aims to explore this mechanism by taking the effect apart and analyzing whether the association between familism and burden may be influenced by another psychosocial variable. In particular, psychosocial influential variables are of interest, as previous research already points to their relevance in associations between familism and wellbeing outcomes, in terms of dysfunctional thoughts and acculturation (Guo et al. 2019; Losada et al. 2010). In this study, we will look at the experience of caregiving gains as possible influential variable.

### The role of positive aspects of care

The appraisal process of caregiving as a possible stressor is highly relevant to the caregiver’s experiences and consequences of the care situation as explained in the previous section (Lazarus and Folkman 2020; Verbakel et al. 2018). In this study, it is assumed that family-centered values could also result in experiencing more *positive aspects of care* (PAC; Carbonneau et al. 2010; Tarlow et al. 2004; Yu et al. 2018).

PAC can encompass feelings of accomplishment and satisfaction about success in caregiving, feelings of personal growth and meaning in life, appreciation of the caregiver–care recipient relationship quality and improved family cohesion and functionality (Carbonneau et al. 2010; Tarlow et al. 2004; Yu et al. 2018). These PAC have shown to be more likely in care contexts that foster role fulfillment and finding meaning in care (Yu et al. 2018). We assume that this is more likely to be experienced among caregivers who endorse family-centered values. Familism values focus the caregiver’s perception and motivation on interconnectedness and loyalty within their family. The care provision for relatives may fulfill these values which is experienced in terms of better family cohesion, functionality and relationship quality, i.e., PAC. However, very few studies have tested the veracity of this assumption in terms of examining the relationship between familism and PAC. Previous studies mostly indicate more resilience, gains and PAC at least among American and British caregivers with higher levels of familism (Falzarano et al. 2022; Parveen and Morrison 2012; Parveen et al. 2014). PAC have also shown to be associated with improved wellbeing of caregivers (Quinn and Toms 2019), for example, higher levels of PAC were connected with lower levels of burden (Abdollahpour et al. 2018; Lee and Li 2022; Quinn and Toms 2019).

### Theoretical framework and aims

**Fig. 1:**
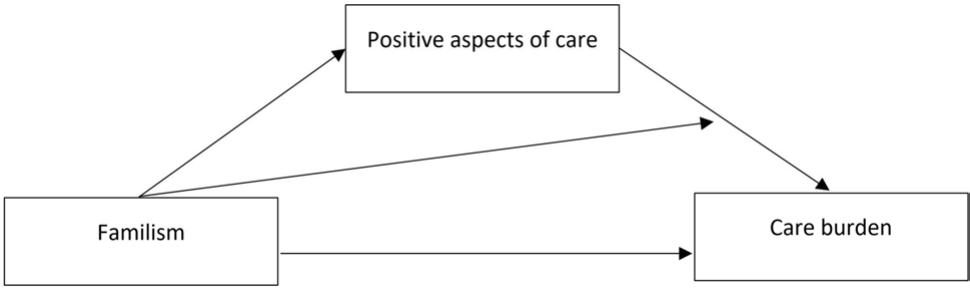
Simplified analytical model tested in this study including the mediation and interaction effect (without the covariates)

Based on previous literature and an extended stress model approach (Knight and Sayegh 2010; Lazarus and Folkman 2020), we assume that higher levels of familism can reduce burden but expect at least a partial mediation by PAC (Fig. 1). Valuing cohesion and reciprocity likely provides a more positive attitude with which the challenges of the care situation are met. This mindset could help to evaluate the caregiving role and situation positively in terms of experiencing satisfaction with the care role and fostering cohesion and reciprocity between caregiver and care recipient; thus, PAC are experienced. This more positive appraisal of the care situation could act as a buffer, resulting in lower levels of burden.

Moreover, this beneficial mediation of familism on burden via PAC may be found only among those, to whom familism values are very important, i.e., who report high levels of familism. Those with low levels of familism values may not experience (the same level of) a protective effect of PAC on burden and may even experience more burden, as indicated in previous literature (Maximiano-Barreto et al. 2022). Thus, not only a mediating but also an (additional) interaction effect may be possible as well and will be tested.

The findings will advance our theoretical understanding of the caregivers’ appraisal mechanisms in the informal care situation and its relevant psychosocial factors. Understanding whether and how the psychosocial factors are associated will also highlight opportunities to intervene and change the appraisal process by the caregiver in order to reduce their care burden. Therefore, this study will test these assumptions by analyzing the mediation effect of PAC and the interaction between familism and PAC in the association between familism and care burden.

## Materials and methods

### Design and sample

The sample was drawn from the ATTIC project, which was designed as a cross-sectional online survey and conducted in December, 8 to 19, 2023 in Germany. Informal (unpaid) caregivers of older adults (≥ 60 years) were sampled from the online access panel of CINT (global market research institution) based on quota to draw a sample representative of informal caregivers, thus oversampling female and middle-age (40 to 64 years) adults. They had to be at least 18 years of age and to provide unpaid care for an older adult (≥ 60 years, relative or friends) regularly for at least six months (e.g., with self-care, household activities, organizational activities). The sample included *n* = 433 who completed the questionnaire, with *n* = 76 being excluded who primarily supported friends or neighbors, resulting in *n* = 357 participants. We focused on informal caregivers of older relatives because familism values were expected to affect in particular caregivers of relatives. Additional attention tests (e.g., participants were asked to choose a specific answer) were conducted, with *n* = 277 succeeding in all tests (*n* = 80 failed in one test), and *n* = 7 had missing values in the question asking about monthly net income. Information on sample size and power calculations is given in the Supplementary Material (Power Calculations; Figure A2). Participants gave written informed consent before starting the survey. Ethics approval was provided by the Ethics Committee of the Center for Psychosocial Medicine of the University Medical Center Hamburg-Eppendorf (LPEK-0575). The project was funded by the Young Academy Fellow individual grant of the Academy of Sciences and Humanities in Hamburg.

### Measurements

*Familism* was measured with the Short Attitudinal Familism Scale (Christophe and Stein 2022). The 6 familism items (e.g., a person should always support members of the extended family, for example, aunts, uncles and in-laws, if they are in need even if it is a big sacrifice; a person should help his or her elderly parents in times of need, for example, helping financially or sharing a house) referred to support, interconnectedness, honor and subjugation (Christophe and Stein 2022; Steidel and Contreras 2003) and were summed up to a mean score (Range: 1 to 10, from strongly disagree to strongly agree), with higher scores indicating stronger belief in familism. The scale was translated by a team of native German and English speakers. Confirmatory factor analysis (CFA) with Satorra–Bentler correction indicated a good model fit (*Χ*^2^(9) = 20.03, *p* < 0.05, RMSA = 0.067, SRMR = 0.037, CFI = 0.978, TLI = 0.964, (Hu and Bentler 1999)), and internal consistency in our sample was good (Cronbach’s alpha 0.88).

*Positive aspects of care* were measured with a German translation of the 9-item PAC scale (Tarlow et al. 2004); translated by a team of native German and English speakers. Participants rated experiences and feelings about the care situation (e.g., feeling needed, useful, appreciated) on a 5-point Likert scale, resulting in a mean score (Range: 1 disagree a lot – 5 agree a lot). Higher scores represent higher levels of positive aspects that the caregivers associate with caregiving. CFA with Satorra–Bentler correction showed a good model fit (*Χ*^2^(27) = 55.87, *p* < 0.001, RMSEA = 0.062, SRMR = 0.036, CFI = 0.974, TLI = 0.965). The scale had excellent reliability (Cronbach’s alpha 0.93) in the analytical sample.

Caregiver *burden* was measured with the German version of the Burden Scale for Family Caregivers short scale (BSFC-s (Graessel et al. 2014)), which measures the appraisal of the care situation as stressful based on 10 items (e.g., wish to run away; caregiving is taking strength; conflicting demands), summed up to a score (Range: 0–30) with higher values indicating higher burden. In this sample, reliability was excellent (Cronbach’s alpha 0.94).

To include personal and contextual factors of relevance for the analyzed associations in line with the stress appraisal approach (Lazarus and Folkman 2020; Verbakel et al. 2018), caregivers were asked about their care situation in terms of using any professional support services (e.g., home care services, self-help groups), intensity of care (hours per week) and whether their care recipient had dementia symptoms and what level of care needs they had. Level of care needs is measured based on the social code book (SGB XI (Federal Ministry of Justice 1994)) with questions asking about mobility, cognitive and communicative abilities, behavioral and psychological problems, ability of self-care and coping independently with impairments, and ability to organize every day and social life (Range: 0 low to 5 high). Information on caregiver’s sociodemographic background was collected including age, gender (male, female, diverse), education (ref. upper secondary school qualification), employment status (ref. employed), marital status (ref. married/in a committed relationship) and monthly net income (categories from < 500€ to ≥ 8000€), and additionally, self-efficacy (Allgemeine Selbstwirksamkeitsskala [General Self-Efficacy Scale], ASKU, mean score, range: 1–5 (Beierlein et al. 2012)) and social support (Oslo Social Support Scale (OSSS-3), sum score, range: 3–14 (Dalgard et al. 2006; Kocalevent et al. 2018)).

### Data analysis

Paired t-tests and Pearson Chi-square tests were conducted to compare the main sample and the subsample of participants failing one attention test. The main analyses were conducted with a conservative approach, including *n* = 277 who succeeded in all attention tests, and additional analyses were conducted repeating the analyses with the full sample.

**Table 1:** Description of the sample (*n* = 277)

	*M* [SD]/*N* (%)
Total	
*Gender*	
Male	95 (34.30)
Female	181 (65.34)
Diverse	1 (0.36)
*Age*	49.37 [13.54]
18–39	77 [27.80]
40–64	143 [51.62]
65+	57 [20.58]
*Marital status*	
Married/in a committed relationship	208 (75.09)
Divorced	26 (9.39)
Widowed	12 (4.33)
Single	31 (11.19)
*Migratory background*	
Direct (participant migrated)	20 (7.22)
Indirect (previous generation migrated)	20 (7.22)
None	237 (85.56)
*Income categories*	
1. below 500€	4 (1.44)
2. 500€ to below 1000€	6 (2.17)
3. 1000€ to below 1500€	18 (6.50)
4. 1500€ to below 2000€	21 (7.58)
5. 2000€ to below 2500€	25 (9.03)
6. 2500€ to below 3000€	27 (9.75)
7. 3000€ to below 3500€	24 (8.66)
8. 3500€ to below 4000€	34 (12.27)
9. 4000€ to below 4500€	22 (7.94)
10. 4500€ to below 5000€	31 (11.19)
11. 5000€ to below 6000€	17 (6.14)
12. 6000€ to below 8000€	17 (6.14)
13. More than 8000€	24 (8.66)
Missing (answer not provided)	7 (2.53)
*Education*	
Upper secondary school	127 (45.85)
Qualification for applied upper secondary school	44 (15.88)
Polytechnic secondary school	18 (6.50)
Intermediate secondary school	64 (23.10)
Lower secondary school	24 (8.66)
*Caregiver-recipient relationship*	
Parents/parents-in-law	144 (51.99)
Grandparents/parents-in-law	66 (23.83)
Partner	43 (15.52)
Other relatives	24 (8.66)
Main caregiver (yes)	211 (76.17)
Use of any form of professional support services for caregiving (Yes)	232 (83.75)
*Level of care needs (Pflegegrad, based on SGB XI)*	
0 (none)	41 (14.80)
1	26 (9.39)
2	85 (30.69)
3	72 (25.99)
4	30 (10.83)
5	23 (8.30)
Care time (hours/week)	15.51 [18.09]
Dementia (Yes)	134 (48.38)
Familism	7.97 [1.54]
Caregiver burden (range: 0–30)	10.41 [7.72]
Positive aspects of care (PAC), range: 1–5	3.81 [0.82]
Self-efficacy (mean score, range: 1–5)	4.06 [0.83]
Social support (range: 3–14)	10 [2.28]

Main caregivers may provide care with others, but they provide the majority of care for the care recipient, supportive caregivers support a main caregiver but provide less care than them; care burden was measured with the Burden Scale for Family Caregivers short scale (BSFC-s, sum score ranging from 0 to 30, higher values indicating higher burden), PAC was measured with the positive aspects of care scale (mean score ranging from 1 to 5, higher values indicating more PAC experiences), familism was measured with the Short Attitudinal Familism Scale (mean score ranging from 1 to 10, higher scores indicating stronger belief in family values),  self-efficacy was measured with the General Self-Efficacy Scale (ASKU, mean score ranging from 1 to 5, higher levels indicating higher self-efficacy), and social support was measured with the Oslo Social Support Scale (OSSS-3, the sum score ranging from 3 to 14 with higher scores indicating more support)

The main analysis included, first, a mediation analysis based on the product method (classic approach, (Baron and Kenny 1986)) with the structural equation modeling command SEM of STATA (Statacorp., Stata Version 18, Texas), using positive aspects of care as mediator, familism as independent and caregiver burden as dependent variable. With outcome and mediator being continuous, the method is equivalent to the differences method (MacKinnon 2008). However, this classic approach cannot account for independent variable (exposure)–mediator interaction, which can lead to biased results if the interaction effect is significant. Thus, a causal mediation analysis within the counterfactual framework was conducted (Pearl 2001; Robins and Greenland 1992; VanderWeele and Vansteelandt 2009). This allows to calculate and decompose the total effect into the natural direct effect (NDE) and natural indirect effect (NIE) and include an exposure–mediator interaction. If this interaction is not significant and the model is correctly specified (i.e., controlling for confounders in both outcome and mediator model), the classic approach and the counterfactual approach both calculate the NDE and NIE (Valeri and Vanderweele 2013). Since we aim to analyze the decomposition of the total effect, the pure natural indirect effect (PNIE) and total natural direct effect (TNDA) will be given as well. Stata version 18 (Stata Corp., Texas) and the mediator command module were used. For the analysis with the counterfactual framework, the familism variable was standardized and one standard deviation below the mean was chosen as control and compared with one standard deviation above the mean. A linear OLS regression model was chosen as method for both outcome and mediator model with robust standard errors. Sociodemographic data, information on the care situation and psychosocial factors relevant to the analyzed associations (Lazarus and Folkman 2020; Verbakel et al. 2018) were included as covariates in the multiple OLS regression analyses of the outcome and mediator model. Cluster-robust standard errors were calculated to account for heteroscedasticity in the data. The level of significance was set at alpha 0.05. Listwise deletion was used to handle missing values, since they only existed for the income variable and their percentage was very low (2.3% of analytical sample). A sensitivity analysis with full-information-maximum-likelihood method assuming missing values are at random (sem, method(mlmv)) was conducted in addition (Enders and Bandalos 2001; Lee and Shi 2021).

## Results

### Description of the sample

The main sample (Table 1) included more women than men (65.34% vs. 34.30%), and participants were on average aged 49.37 years (SD = 13.54, range: 20–83 years). Only few had a migratory background, with 7.22% having migrated themselves and 7.22% having parents who migrated to Germany. The majority of the caregivers were married or in a committed relationship (75.09%) and had either an upper secondary school (45.85%) or an intermediate secondary school qualification (23.10%). A median of 8 (3500€ to below 4000€) and an interquartile range between 5 and 10 (IQR = 5 (2000€ to below 2500€) – 10 (4500€ to below 5000€)) of the monthly net income were found; thus, half of the sample earned between 2000 and 5000 Euro per month. About 52% of the sample provided care for their parents or parents-in-law and most used some form of formal care support (83.75%). The majority of caregivers reported to provide for care recipients with a care dependency of level 2 (30.69%) or 3 (25.99%) (average level of care needs: *M* = 2.33, SD = 1.42), and 48.38% reported their care recipients to show dementia symptoms. They provided on average 15.51 h of care per week but variation was large (SD = 18.09). The sample reported a care burden of on average 10.41 (SD = 7.72); agreement with familism values was on average 7.97 (SD = 1.54) and experience of positive aspects of care was on average 3.81 (SD = 0.82).

The participants failing the attention tests (*n* = 80, Table A1, Supplementary Material) differed from the main sample in terms of being significantly younger (M = 43.48, SD = 14.16, *t*(355) = − 3.39, *p* < 0.001) and more of these participants provided care for relatives with dementia (65%, Pearson *Χ*^2^ (1) = 6.87, *p* < 0.01). They also had higher levels of burden (M = 13.11, SD = 7.42, *t*(355) = 2.78, *p* < 0.01) and lower levels of positive aspects of care (M = 3.57, SD = 0.83, t(355) = − 2.32, *p* < 0.05). The samples also showed a significant difference in education level (Pearson *Χ*^2^(5) = 13.34, *p* < 0.05), with the main sample having less participants with a polytechnic secondary school degree but more participants with any of the other degrees compared to the subsample. No differences between samples were found in the other variables.

### Findings of main analysis

Findings with the classic mediation approach (Model 1, Table 2 and Supplementary Material Figure A3) showed a significant direct effect (*b* = 0.99, *p* < 0.01) between familism and burden, between PAC and burden (*b* = −2.92, *p* < 0.001) and a significant indirect effect of familism through PAC on burden (*b* = − 0.64, *p* < 0.01). The total effect was not significant (*b* = 0.35, *p* = 0.239).

**Table 2:** Results of classic mediator analysis (Model 1) and causal mediation models (Models 2 and 3)

Model	*b*	SE_robust_	*p*	CI
*Model 1 (SEM)*				
Direct effect (familism → burden)	0.99	0.32	0.002	[0.38; 1.61]
Direct effect (PAC → burden)	– 2.92	0.76	0.000	[− 4.41; − 1.43]
Direct effect (familism → PAC)	0.22	0.04	0.000	[0.15; 0.29]
Indirect effect (familism → PAC → burden)	−0.64	0.20	0.001	[− 1.03; − 0.25]
Total effect	0.35	0.30	0.239	[− 0.23; 0.94]
*Model 2*				
Natural direct effects (NDE)	3.12	0.99	0.002	[1.17; 5.06]
Natural indirect effect (NIE)	− 1.60	0.63	0.011	[− 2.84; −0.37]
Total natural direct effect (TNDE)	3.18	1.17	0.007	[0.89; 5.48]
Pure natural indirect effect (PNIE)	− 1.67	0.56	0.003	[− 2.75; −0.58]
Total effect (TE)	1.52	1.07	0.157	[− 0.59; 3.62]
Exposure × Mediator interaction (familism × PAC)	0.06	0.44	0.900	[− 0.81; 0.92]
*Model 3*				
NIE				
(1 vs. 0) (reference mean value vs. − 2 SD)	1.70	0.66	0.011	[0.40; 3.00]
(2 vs. 0) (reference mean value vs. − 1 SD)	0.83	0.28	0.003	[0.29; 1.37]
(3 vs. 0) (reference mean value vs. + 1 SD)	− 0.80	0.32	0.012	[− 1.42; − 0.18]
(4 vs. 0) (reference mean value vs. + 2 SD)	− 1.57	0.79	0.048	[− 3.13; − 0.01]
NDE				
(1 vs. 0) (reference mean value vs. − 2 SD)	− 3.15	1.06	0.003	[-5.22; − 1.08]
(2 vs. 0) (reference mean value vs. − 1 SD)	− 1.58	0.53	0.003	[− 2.61; − 0.54]
(3 vs. 0) (reference mean value vs. + 1 SD)	1.58	0.53	0.003	[.54; 2.61]
(4 vs. 0) (reference mean value vs. + 2 SD)	3.15	1.06	0.003	[1.08; 5.22]
*TE*				
(1 vs. 0) (reference mean value vs. − 2 SD)	− 1.45	0.96	0.128	[− 3.33; 0.42]
(2 vs. 0) (reference mean value vs. − 1 SD)	− 0.74	0.49	0.131	[− 1.71; 0.22]
(3 vs. 0) (reference mean value vs. + 1 SD)	0.77	0.60	0.200	[− 0.41; 1.96]
(4 vs. 0) (reference mean value vs. + 2 SD)	1.58	1.37	0.249	[− 1.11; 4.26]
PAC × familism	0.06	0.44	0.900	[− 0.81; 0.92]

All models controlled for self-efficacy, social support, age, gender (ref. male), education (ref. upper secondary school qualification), employment status (ref. employed), marital status (ref. married/in a committed relationship/partnership), monthly net income (< 500€, 500 to < 1000€, 1000 to < 1500€, 1500 € to < 2000 €, 2000 € to < 2500 €, 2500 € to < 3000€, 3000€ to < 3500€, 3500€ to < 4000 €, 4000€ to < 4500€, 4500€ to < 5000€, 5000€ to < 6000€, 6000€ to < 8000€, ≥ 8000€), use of professional support services (ref. no), care recipient with dementia symptoms (ref. no), care time (hours per week), level of care needs (0–5), with Models 2 and 3 controlling these in the outcome and mediator model; linear OLS regression with robust standard errors was calculated. Model 1 was conducted with SEM (classic approach, product method), Models 2 and 3 were conducted with the mediator module from Statacorp (causal mediation analysis allowing for exposure–mediator interaction), Model 2 used a standardized familism variable with M minus 1 SD as control (and M plus 1 SD as comparison group), Model 3 used the mean as comparison group to M minus and plus 1 and 2 SD

**Fig. 2:**
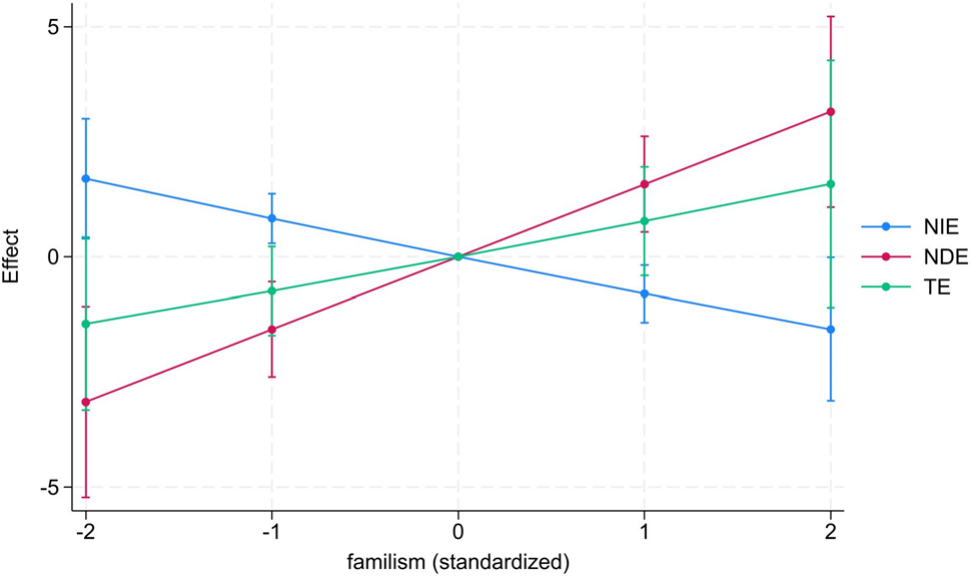
Illustration of the results of the natural indirect effect (NIE), the natural direct effect (NDE) and the total effect (TE) based on Model 3 (*n* = 277) using the mean value of the standardized familism (treatment) variable as comparison group for values of 1 or 2 standard deviations (SD) above or below the mean (M)

The causal mediation analysis also estimated a significant direct effect (NDE) between familism and burden (*b* = 3.12, *p* < 0.01) and an indirect effect through PAC (NIE, *b* = -1.60, *p* < 0.05). The total effect was not significant (*b* = 1.52, *p* = 0.157). Adding to this, the pure natural indirect effect (PNIE, *b* = -1.67, *p* < 0.01) and the total natural direct effect (TNDE, *b* = 3.18, *p* < 0.01) were significant. The model allowed for interaction between familism and PAC, but the interaction was not significant (*b* = 0.06, *p* = 0.900). An additional model was calculated using the mean as reference for comparison with values one and two standard deviations below and above the mean (Table 1, model 3) with the findings illustrated in Fig. 2.

### Findings of additional analyses

Findings for the FIML analysis of the main sample (Supplementary Material, Table A2) showed a significant direct effect (*b* = 0.99, *p* < 0.01) between familism and burden, between PAC and burden (*b* = -2.92, *p* < 0.001) and a significant indirect effect of familism through PAC on burden (*b* = − 0.64, *p* < 0.01) again. The total effect was not significant (*b* = 0.35, *p* = 0.239).

Findings of the additional analysis using the classic approach with the full sample (Supplementary Material, Table A3, Model 1) found a significant direct effect (*b* = 1.15, *p* < 0.001) between familism and burden, between PAC and burden (*b* = -2.95, *p* < 0.001) and a significant indirect effect of familism through PAC on burden (*b* = − 0.57, *p* < 0.001). The total effect was now significant (*b* = 0.58, *p* < 0.05). The causal mediation analysis with the full sample (Supplementary Material, Table A3, Model 2) also found a significant NDE between familism and burden (*b* = 3.57, *p* < 0.001) and a significant NIE through PAC (*b* = − 1.44, *p* < 0.01). The TNDE (*b* = 3.54, *p* < 0.001) and PNIE (*b* = -1.41, *p* < 0.01) were significant as well, while the exposure–mediator interaction was not significant (*b* = − 0.03, *p* = 0.939).

## Discussion

This study aimed to analyze whether the association between family-centered values (i.e., familism) and informal care burden is at least partially mediated by positive experiences of care (i.e., PAC). The findings of all analyzed models provide support for this association and provide new insights into the mechanisms between these constructs. This extends our previous understanding and highlights the important role of PAC in the stress appraisal process based on the informal caregiver’s values.

Both the classic and the causal mediation approach showed that higher levels of familism were associated with higher levels of burden. This is in contrast with some of the previous studies’ findings (Guo et al. 2019; Gupta et al. 2009; Maximiano-Barreto et al. 2022). A possible explanation could be that the familism values can also be perceived as obligation and duty by the caregiver, which may exacerbate the perceived demands regarding care, in line with the stress appraisal process approach (Lazarus and Folkman 2020; Verbakel et al. 2018). The findings of this study confirm this by showing that burden is perceived to be higher among those endorsing more family values. These findings add to previous research that found familism to be associated with worse mental health (Tian et al. 2022) and dysfunctional thoughts (Losada et al. 2010). Moreover, our findings on the role of PAC in this association provide a possible explanation for the conflicting findings in previous research regarding the association between familism and burden, and are in line with our assumptions.

The direct effect of familism on burden was partially mediated by PAC. In line with previous findings, PAC was associated with higher familism values (Falzarano et al. 2022; Parveen and Morrison 2012; Parveen et al. 2014) and was associated with lower burden (Abdollahpour et al. 2018; Lee and Li 2022; Quinn and Toms 2019). Thus, previous studies’ findings pointing to familism as a protective factor (Guo et al. 2019; Gupta et al. 2009; Maximiano-Barreto et al. 2022) may in fact have found the hidden mediating (protective) effect of PAC. Once PAC is taken into account as a mediator, the direct effect of familism on burden is pointing to these values as a risk instead, i.e., more familism resulting in more burden. Findings for all effects, including findings of the classic approach, also showed that the direct negative effect of familism on burden is larger than the indirect effect via PAC. While one effect is positive, the other is negative, resulting in a smaller and non-significant total effect. This is in line with a recent meta-analysis not finding a significant association at all (Tian et al. 2022). Thus, our findings confirm and extend previous results by showing that the non-significant association seems to be the result of the mediating (protective) and direct (risk) effect balancing and canceling each other out. The findings thus indicate that fostering more PAC can help to reduce the negative effect of familism on burden further, while loss of PAC could instead increase the risk of familism for burden again. As previous research had not considered the role of PAC in this context and overlooked these mechanisms, our findings fill an important research gap by confirming its role and its relevance.

This study also analyzed the exposure–mediator interaction within the causal mediation model. It was not significant in our analysis; thus, the findings of the classic and the causal mediation approach both calculated the NDE and NIE (Valeri and Vanderweele 2013), as shown when comparing our models. Differences in the estimates are due to calculating the causal mediation model with a standardized measure of familism. The causal mediation approach allowed us to calculate the PNIE and TNDE in addition to the NDE and NIE, which enabled us to focus on how the total effect can be decomposed. All main models clearly indicated a significant negative direct and significant positive indirect effect as well as a non-significant total effect. While this confirms that familism is of relevance to burden, the findings also point out that PAC are a priority in regard to reducing burden. This effect was found irrespective of the level of familism; thus, all caregivers—with high or low levels of familism—can benefit from PAC and this is the primary factor reducing burden, not familism, as previous studies indicated (Guo et al. 2019; Gupta et al. 2009; Maximiano-Barreto et al. 2022).

Additional analyses with FIML resulted in the same estimates as the classic and causal mediation models using listwise deletion. However, findings were different in the analysis including the participants, who had been dropped due to failing in all attention tests in the questionnaire (full sample). This group of caregivers, which had been dropped from the main analyses, was on average younger, more burdened, experienced less PAC and more of them provided care for relatives with dementia. The mediation effect of familism on burden by PAC was still found. However, the direct effect of familism on burden was larger and the indirect effect through PAC could not fully offset it. Thus, familism still had a worsening impact on burden, i.e., increasing it, although part of this effect was mediated and reduced through PAC. A reason for the difference in the total effect to the findings in the main analysis could be the inclusion of more dementia caregivers. Although we controlled for dementia care and intensity of care in terms of hours of care per week and level of care needs in our models, further aspects of dementia care (e.g., type of symptoms) could be relevant (Lindt et al. 2020). Further research on subgroups of informal caregivers for older adults with different reasons of care needs (i.e., different illnesses) is recommended. Also, additional testing (Table A4) with subgroups of informal caregivers (male and female caregivers, main and supportive caregivers, caregivers with and without migratory background) indicates the effects can be found for all tested groups. However, some of the subgroups were rather small; thus, the findings should be viewed with great caution. Further research on these groups with larger sample sizes is recommended to add to our findings.

Limiting our findings is the cross-sectional study design. Causal conclusions based on our models are dependent on the model including all relevant confounding variables and being correctly specified (Valeri and Vanderweele 2013). Future research with a longitudinal design is recommended to add to our findings.

## Conclusion

In sum, the findings highlight the relevance of PAC for the informal care appraisal process and the importance of taking mediating factors into account when analyzing the association between familism values and burden. The findings implicate that while values are important for the appraisal process in caregiving and its outcome in terms of burden, fostering familism would not be helpful to reduce burden, unless these values are supporting PAC. Thus, fostering PAC is indicated to be important for reducing burden and can help all caregivers, irrespective of their level of familism values. It is therefore recommended that more attention is given to supporting positive experiences of care by including them into care models and concepts. Further research should focus on identifying the factors relevant for supporting positive experiences of care and on their role in transforming familism values into PAC, for example, in terms of role fulfilment and satisfaction with care. This could add to our findings and help advance the theory of caregiver resilience mechanisms further. Moreover, considering the increasing demand for informal care with ongoing population aging, in particular in Germany (Federal Statistical Office 2020; Statistisches Bundesamt 2020), greater efforts are needed to adapt the care system to provide structural support that promotes positive experiences of caregiving for relatives.

### Supplementary Information

Below is the link to the electronic supplementary material.

Supplementary file1 (DOCX 66 kb)

## Data Availability

The data that support the findings of this study are available from the corresponding author upon reasonable request.
