# Supplementary material for: Look on the bright side: the relation between family values, positive aspects of care and caregiver burden
Source: Eur J Ageing. 2024 Aug 30;21(1):23. doi: 10.1007/s10433-024-00819-9 (PMC11364734; doi:10.1007/s10433-024-00819-9)
Supplement: Supplementary file 1 — Supplementary file1 (DOCX 66 kb) [file 10433_2024_819_MOESM1_ESM.docx]

**Supplementary Material for Manuscript “Look on the bright side: The relation between family values, positive aspects of care and caregiver burden”**

**Power calculations**

Sample size in this project had been chosen based on literature recommendations for validation sample sizes (Hu & Bentler, 1999; MacCallum et al., 2001) and power calculations for multiple regression analysis analyzing effects of medium size (power (1-β= 0.95, level of significance α =0.05, up to 12 predictors; calculated with GPower Version 3.1.9.7; (Faul et al., 2009; Faul et al., 2007)) which would require about 200 participants.

Sample size calculation for mediation analysis is more complex. Based on Sim et al (2022), our sample size of n=277 is sufficient to analyze effects of medium size (focus is on the indirect effect) with the path model we tested with the SEM command from Stata. However, power analysis for causal mediation analysis is still understudied, in particular for studies using continuous treatment, mediator and outcome variables (Qin, 2024b; VanderWeele, 2020). Easy-to-use packages like GPower are not available for this approach (VanderWeele, 2020). Thus, we used the approach and online app from Qin (Qin, 2024a, 2024b) to calculate power based on our available sample size. We found our sample to be sufficient to analyze medium sized indirect effects with causal mediation models (NIE: Power 1-β=.96 for n=277 and 1-β=.55 for n=100; PNIE: Power 1-β=.99 for n=277 and 1-β=.65 for n=100).

**References for power calculations**

Faul, F., Erdfelder, E., Buchner, A., & Lang, A.-G. (2009). Statistical power analyses using G* Power 3.1: Tests for correlation and regression analyses. *Behavior Research Methods*, *41*(4), 1149-1160.

Faul, F., Erdfelder, E., Lang, A.-G., & Buchner, A. (2007). G* Power 3: A flexible statistical power analysis program for the social, behavioral, and biomedical sciences. *Behavior Research Methods*, *39*(2), 175-191.

Hu, L. T., & Bentler, P. M. (1999). Cutoff Criteria for Fit Indexes in Covariance Structure Analysis: Conventional Criteria Versus New Alternatives. *Structural Equation Modeling-a Multidisciplinary Journal*, *6*(1), 1-55. https://doi.org/10.1080/10705519909540118

MacCallum, R. C., Widaman, K. F., Preacher, K. J., & Hong, S. (2001). Sample Size in Factor Analysis: The Role of Model Error. *Multivariate Behav Res*, *36*(4), 611-637. https://doi.org/10.1207/S15327906MBR3604_06

Qin, X. (2024a). Correction to: Sample size and power calculations for causal mediation analysis: A tutorial and Shiny app. *Behavior Research Methods*, *56*(4), 4216-4216. https://doi.org/10.3758/s13428-024-02386-4

Qin, X. (2024b). Sample size and power calculations for causal mediation analysis: A Tutorial and Shiny App. *Behavior Research Methods*, *56*(3), 1738-1769. https://doi.org/10.3758/s13428-023-02118-0

VanderWeele, T. J. (2020). Invited Commentary: Frontiers of Power Assessment in Mediation Analysis. *American Journal of Epidemiology*, *189*(12), 1568-1570. https://doi.org/10.1093/aje/kwaa081

**Tables**

Table A1

Description of main sample (n=277), subsample (participants failing at one attention tests, n=80) and of full sample (n= 357 including main sample and subsample)

|  | M[SD] /N(%) | | | |
| --- | --- | --- | --- | --- |
|  | Main sample | Subsample | *p* | Full sample |
| Total | 277 | 80 |  | 357 |
| Gender |  |  | ns |  |
| - male | 95 (34.30) | 22 (27.50) |  | 117 (32.77) |
| - female | 181 (65.34) | 58 (72.50) |  | 239 (66.95) |
| - diverse | 1 (0.36) | 0 (0) |  | 1 (0.28) |
| Age | 49.37 [13.54] | 43.48 [14.16] | <.001 | 48.05 [13.88] |
| Age group |  |  | <.01 |  |
| - 18-39 | 77 (27.80) | 37 (46.25) |  | 114 (31.94 |
| - 40-64 | 143 (51.62) | 35 (43.75) |  | 178 (49.86) |
| - 65+ | 57 (20.58) | 8 (10.00) |  | 65 (18.21) |
| Marital status |  |  | ns |  |
| - married/in a committed relationship | 208 (75.09) | 59 (73.75) |  | 267 (74.79) |
| - divorced | 26 (9.39) | 4 (5.00) |  | 30 (8.40) |
| - widowed | 12 (4.33) | 2 (2.50) |  | 14 (3.92) |
| - single | 31 (11.19) | 15 (18.75) |  | 46 (12.89) |
| Migratory background |  |  | ns |  |
| - direct (participant migrated) | 20 (7.22) | 3 (3.75) |  | 23 (6.44) |
| - indirect (previous generation migrated) | 20 (7.22) | 11 (13.75) |  | 31 (8.68) |
| - none | 237 (85.56) | 66 (82.50) |  | 303 (84.87) |
| Income categories |  |  | ns |  |
| - below 500€ | 4 (1.44) | 0 (0) |  | 4 (1.12) |
| - 500€ to below 1,000€ | 6 (2.17) | 1 (1.25 ) |  | 7 (1.96) |
| - 1,000€ to below 1,500€ | 18 (6.50) | 6 (7.50) |  | 24 (6.72) |
| - 1,500€ to below 2,000€ | 21 (7.58) | 8 (10.00) |  | 29 (8.12) |
| - 2,000€ to below 2,500€ | 25 (9.03) | 8 (10.00) |  | 33 (9.24) |
| - 2,500€ to below 3,000€ | 27 (9.75) | 9 (11.25) |  | 36 (10.08) |
| - 3,000€ to below 3,500€ | 24 (8.66) | 2 (2.50) |  | 26 (7.28) |
| - 3,500€ to below 4,000€ | 34 (12.27) | 6 ( 7.50) |  | 40 (11.20) |
| - 4,000€ to below 4,500€ | 22 (7.94) | 6 (7.50) |  | 28 (7.84) |
| - 4,500€ to below 5,000€ | 31 (11.19) | 6 (7.50) |  | 37 (10.36) |
| - 5,000€ to below 6,000€ | 17 (6.14) | 13 (16.25) |  | 30 (8.40) |
| - 6,000€ to below 8,000€ | 17 (6.14) | 9 (11.25) |  | 26 (7.28) |
| - more than 8,000€ | 24 (8.66) | 5 (6.25) |  | 29 (8.12) |
| - missing (answer not provided) | 7 (2.53) | 1 (1.25) |  | 8 (2.24) |
| Education |  |  | <.05 |  |
| - upper secondary school | 127 (45.85) | 32 (40.00) |  | 159 (44.54) |
| - qualification for applied upper secondary school | 44 (15.88) | 3 (3.75) |  | 21 (5.88) |
| - polytechnic secondary school | 18 (6.50) | 18 (22.50) |  | 82 (22.97) |
| - intermediate secondary school | 64 (23.10) | 6 (7.50) |  | 30 (8.40) |
| - lower secondary school | 24 (8.66) | 3 (3.75) |  | 3 (0.84) |
| Caregiver-recipient relationship |  |  | ns |  |
| - parents/parents-in-law | 144 (51.99) | 42 (52.50) |  |  |
| - grandparents/parents-in-law | 66 (23.83) | 20 (25.00) |  |  |
| - partner | 43 (15.52) | 9 (11.25) |  |  |
| - other relatives | 24 (8.66) | 9 (11.25) |  |  |
| Main caregiver (yes) | 211 (76.17) | 69 (86.25) | ns | 280 (78.43) |
| Use of any form of professional support services for caregiving (Yes) | 232 (83.75) | 71 (88.75) | ns | 303 (84.87) |
| Level of care needs (Pflegegrad, based on SGB XI) |  |  | ns |  |
| - 0 (none) | 41 (14.80) | 11 (13.75) |  | 52 (14.57) |
| - 1 | 26 (9.39) | 9 (11.25) |  | 35 (9.80) |
| - 2 | 85 (30.69) | 21 (26.25) |  | 106 (29.69) |
| - 3 | 72 (25.99) | 18 (22.50) |  | 90 (25.21) |
| - 4 | 30 (10.83) | 16 (20.00) |  | 46 (12.89) |
| - 5 | 23 (8.30) | 5 (6.25) |  | 28 (7.84) |
| Care time (hours/week) | 15.51 [18.09] | 14.75 [ 13.65] | ns | 15.34 [17.18] |
| Dementia (Yes) | 134 (48.38) | 52 (65.00) | <.01 | 186 (52.10) |
| Familism | 7.97 [1.54] | 7.63 [1.68] | ns | 7.89 [1.57] |
| Caregiver burden (Range: 0-30) | 10.41 [7.72] | 13.11 [7.42] | <.01 | 11.02 [7.73] |
| Positive aspects of care (PAC), Range: 1-5 | 3.81 [.82] | 3.57 [.83] | <.05 | 3.75 [.82] |
| Self-efficacy (mean score, Range: 1-5) | 4.06 [.83] | 3.9 [.75] | ns | 4.03 [.82] |
| Social support (Range: 3-14) | 10 [2.28] | 9.59 [2.24] | ns | 9.91 [2.28] |

*Note.* Care burden was measured with the Burden Scale for Family Caregivers short scale (BSFC-s, sum score ranging from 0 to 30, higher values indicating higher burden), PAC was measured with the positive aspects of care scale (mean score ranging from 1 to 5, higher values indicating more PAC experiences), self-efficacy was measured with the General Self-Efficacy Scale (ASKU, mean score ranging from 1 to 5, higher levels indicating higher self-efficacy) and social support was measured with the Oslo Social Support Scale (OSSS-3, the sum score ranging from 3 to 14 with higher scores indicating more support). Paired t-tests and Pearson *Χ*^2^ tests were conducted to compare the main and subsample.

Table A2

Mediation model with full-information maximum likelihood

| Model | b | SE_robust_ | *p* | CI |
| --- | --- | --- | --- | --- |
| **Model 1** (SEM) |  |  |  |  |
| Direct effect (familism 🡪 burden) | .99 | .32 | 0.002 | [.38; 1.61] |
| Direct effect (PAC 🡪 burden) | -2.92 | .76 | 0.000 | [-4.41; -1.43] |
| Direct effect (familism 🡪 PAC) | .22 | .04 | 0.000 | [.15; .29] |
| Indirect effect (familism 🡪 PAC 🡪 burden) | -.64 | .20 | 0.001 | [-1.03; -.25] |
| Total effect | .35 | .30 | 0.239 | [-.23; .94] |

*Note*. Model was controlled for self-efficacy, social support, age, gender (ref. male), education (ref. upper secondary school qualification), employment status (ref. employed), marital status (ref. married/in a committed relationship/partnership), monthly net income (500 to ≥8000€), use of professional support services (ref. no), care recipient with dementia symptoms (ref. no), care time (hours per week), level of care needs (0-5); conducted with SEM (classic approach, product method), unstandardized coefficients and cluster-robust standard errors are given.

Table A3

Results of classic mediator analysis (Model 1) and causal mediation models (Model 2) with the full sample (n=357)

| Model | b | SE_robust_ | *p* | CI |
| --- | --- | --- | --- | --- |
| **Model 1** |  |  |  |  |
| Direct effect (familism 🡪 burden) | 1.15 | .28 | <.001 | [.61; 1.69] |
| Direct effect (PAC 🡪 burden) | -2.95 | .64 | <.001 | [-4.21; -1.70] |
| Direct effect (familism 🡪 PAC) | .19 | .03 | <.001 | [.13; .25] |
| Indirect effect (familism 🡪 PAC 🡪 burden) | -.57 | .15 | <.001 | [-.87; -.28] |
| Total effect | .58 | .27 | .030 | [.06; 1.10] |
| **Model 2** |  |  |  |  |
| Natural Direct Effects (NDE) (familism 🡪 burden) | 3.57 | .87 | <.001 | [1.86; 5.28] |
| Natural Indirect Effect (NIE) (familism 🡪 PAC 🡪 burden) | -1.44 | .49 | 0.003 | [-2.41; -.48] |
| Total Natural Direct Effect (TNDE) | 3.54 | 1.01 | <.001 | [1.57; 5.51] |
| Pure Natural Indirect Effect (PNIE) (familism 🡪 PAC 🡪 burden) | -1.41 | .43 | 0.001 | [-2.26; -.57] |
| Total Effect (TE) | 2.13 | .93 | 0.022 | [.31; 3.94] |
| Exposure x Mediator Interaction (familism x PAC) | -.03 | .41 | 0.939 | [-.83; .77] |

*Note*. All models controlled for self-efficacy, social support, age, gender (ref. male), education (ref. upper secondary school qualification), employment status (ref. employed), marital status (ref. married/in a committed relationship/partnership), monthly net income (500 to ≥8000€), use of professional support services (ref. no), care recipient with dementia symptoms (ref. no), care time (hours per week), level of care needs (0-5), with Model 2 controlling these in the outcome and mediator model; linear OLS regression with robust standard errors were calculated. Model 1 was conducted with SEM (classic approach, product method), Models 2 was conducted with the mediator module from Statacorp. (causal mediation analysis) and used a standardized familism variable with M minus 1 SD as control (and M plus 1 SD as comparison group).

Table A4

Additional subgroup analysis with structural equation model approach (models 1) and with causal mediation approach (models 2)

|  | Male CG |  |  |  | Female CG |  |  |  | Main CG |  |  |  | Supportive CG |  |  |  | CG with migratory background |  |  |  | CG without migratory background |  |  |  |
| --- | --- | --- | --- | --- | --- | --- | --- | --- | --- | --- | --- | --- | --- | --- | --- | --- | --- | --- | --- | --- | --- | --- | --- | --- |
| *N* | 95 |  |  |  | 181 |  |  |  | 211 |  |  |  | 66 |  |  |  | 40 |  |  |  | 237 |  |  |  |
| Model | b | SE_robust_ | p | CI | b | SE_robust_ | p | CI | b | SE_robust_ | p | CI | b | SE_robust_ | p | CI | b | SE_robust_ | p | CI | b | SE_robust_ | p | CI |
| **Model 1** (SEM) |  |  |  |  |  |  |  |  |  |  |  |  |  |  |  |  |  |  |  |  |  |  |  |  |
| Direct effect (familism 🡪 burden) | 1.87 | .42 | 0.000 | 1.05; 2.70 | .84 | .41 | 0.040 | .04; 1.65 | .92 | .36 | 0.012 | .20; 1.63 | 1.09 | .44 | 0.014 | .22; 1.96 | 1.00 | 1.62 | 0.535 | -2.17; 4.17 | 1.10 | .34 | 0.001 | .42; 1.78 |
| Direct effect (PAC 🡪 burden) | -3.52 | .70 | 0.000 | -4.88; -2.15 | -3.08 | 1.20 | 0.010 | -5.43; -.73 | -2.79 | .88 | 0.001 | -4.51; -1.07 | -3.54 | .99 | 0.000 | -5.48; -1.60 | -7.91 | 3.69 | 0.032 | -15.13; -.69 | -2.81 | .81 | 0.001 | -4.40; -1.22 |
| Direct effect (familism 🡪 PAC) | .31 | .06 | 0.000 | .18; .44 | .19 | .04 | 0.000 | .12; .27 | .24 | .04 | 0.000 | .15; .32 | .18 | .05 | 0.001 | .07; .29 | .09 | .07 | 0.199 | -.05; .22 | .23 | .04 | 0.000 | .15; .31 |
| Indirect effect (familism 🡪 PAC 🡪 burden) | -1.09 | .34 | 0.001 | -1.75; -.43 | -.60 | .26 | 0.020 | -1.10; -.09 | -.66 | .24 | 0.006 | -1.13; -.19 | -.64 | .22 | 0.003 | -1.06; -.21 | -.70 | .59 | 0.238 | -1.85; .46 | -.64 | .22 | 0.004 | -1.07; -.20 |
| Total effect | .78 | .39 | 0.044 | .02; 1.55 | .25 | .40 | 0.541 | .18; .44 | .26 | .33 | 0.440 | -.40; .91 | .45 | .51 | 0.372 | -.54; 1.44 | .31 | 1.80 | 0.864 | -3.22; 3.84 | .47 | .32 | 0.149 | -.17; 1.10 |
| **Model 2** (CMM) |  |  |  |  |  |  |  |  |  |  |  |  |  |  |  |  |  |  |  |  |  |  |  |  |
| Natural Direct Effects (NDE) (familism 🡪 burden) | 5.76 | 1.32 | 0.000 | 3.18; 8.35 | 2.66 | 1.30 | 0.040 | .12; 5.20 | 2.92 | 1.17 | 0.013 | .63; 5.22 | 3.43 | 1.42 | 0.016 | .64; 6.21 | - |  |  |  | 3.55 | 1.07 | 0.001 | 1.45; 5.65 |
| Natural Indirect Effect (NIE) (familism 🡪 PAC 🡪 burden) | -3.33 | 1.35 | 0.013 | -5.96; -.69 | -1.37; -2.00 | .69 | 0.046 | -2.72; -.03 | -1.65 | .76 | 0.030 | -3.13; -.16 | -1.52 | .70 | 0.030 | -2.90; -.15 | - |  |  |  | -1.43 | .70 | 0.042 | -2.81; -.05 |
| Total Effect (TE) | 2.44 | 1.67 | 0.144 | -.84; 5.71 | 1.29; 0.96 | 1.34 | 0.335 | -1.335167 3.915578 | 1.28 | 1.26 | 0.309 | -1.19; 3.74 | 1.90 | 1.64 | 0.247 | -1.32; 5.12 | - |  |  |  | 2.13 | 1.12 | 0.058 | -.07; 4.32 |
| Exposure x Mediator Interaction (familism x PAC) | -.19 | .38 | 0.616 | -.93; .55 | .09; 0.14 | .67 | 0.891 | -1.223787 1.406944 | .13 | .50 | 0.798 | -.85; 1.11 | -.10 | .77 | 0.892 | -1.62; 1.41 | - |  |  |  | .29 | .46 | 0.525 | -.61; 1.20 |

*Notes*. CG = caregiver; main caregivers may provide care with others but they provide the majority of care for the care recipient, supportive caregivers support a main caregiver but provide less care than them, caregivers with migratory background either migrated themselves or have at least one parent who migrated, caregivers without migratory background have not made any migration experiences and neither do they have parents who did; the causal mediation model for the group of caregivers with migratory background lacked sufficient sample size to be conducted and the structural equation model likely provides biased estimates due to this.

**Figures**

Figure A1

Stressor

(e.g., informal caregiving)

Appraisal process

Short & long-term outcomes

(e.g., burden, wellbeing)

Contextual or personal
barriers/demands and resources
(*~ moderating and mediating factors*)

(e.g., familism)

Figure legend. Simplified depiction of the care-related stress model that serves as theoretical basis of this study

Figure A2

n=1386

Screenout (n=789)

- n=639 filtered as non-caregivers or did not provide informed consent
- n=150 quality screenouts (answered >1 quality question wrong)

Dropout (n=164 incomplete interviews)

n=433 complete interviews

n=76 caregivers of friends or neighbors excluded

n=277 informal caregivers of older relatives (*Main Sample*)

n=357 informal caregivers of older relatives (*Full Sample*)

n=80 dropped for failing in one attention test

Figure legend: Flow of sample selection

Figure A3

Positive aspects of care

Familism

Care burden

-2.92***

.22***

.99**

Figure legend: Model 1 calculated with Structural Equation Modelling (SEM) of the mediation between familism and burden by positive aspects of care (covariates are not depicted).
